# An occurence records database of French Guiana harvestmen (Arachnida, Opiliones)

**DOI:** 10.3897/BDJ.2.e4244

**Published:** 2014-12-25

**Authors:** Sébastien Cally, Pierre Solbès, Bernadette Grosso, Jérôme Murienne

**Affiliations:** † UMR5174 CNRS/UPS/ENFA, Toulouse, France

**Keywords:** Occurrence, French Guiana, Neotropics, Opiliones

## Abstract

This dataset provides information on specimens of harvestmen (Arthropoda, Arachnida, Opiliones) collected in French Guiana. Field collections have been initiated in 2012 within the framework of the CEnter for the Study of Biodiversity in Amazonia (CEBA: www.labex-ceba.fr/en/). This dataset is a work in progress.  Occurrences are recorded in an online database stored at the EDB laboratory after each collecting trip and the dataset is updated on a monthly basis. Voucher specimens and associated DNA are also stored at the EDB laboratory until deposition in natural history Museums. The latest version of the dataset is publicly and freely accessible through our Integrated Publication Toolkit at http://130.120.204.55:8080/ipt/resource.do?r=harvestmen_of_french_guiana or through the Global Biodiversity Information Facility data portal at http://www.gbif.org/dataset/3c9e2297-bf20-4827-928e-7c7eefd9432c.

## Introduction

French Guiana is a French overseas department covered at 97% by primary forest (Gond et al. 2011). As such, it represents the only Outermost Region of Europe covering a large fraction of mainland in tropical South America. The territory is part of the Guianan moist forests ecoregion and has strong biogeographic affinities with the rest of the Amazonia bioregion (Olson et al. 2001). The biological diversity of French Guiana is now one of the best known in South America, yet some gaps in knowledge still exist, especially in arthropods.

With more than 6500 species, harvestmen (Opiliones) is the third most diverse order of arachnid after Acari and Araneae (Kury 2013). They occur in superficial soil layers, leaf litter, under bark, and on vegetation from ground to canopy (Pinto-da-Rocha and Bonaldo 2006). The maximum richness of harvestmen species is found in the Neotropical region, however, diversity patterns within this region are still poorly known, both on regional and local scales. Because of their relatively high abundance and diversity in the Neotropical forests, they are useful candidates for studying community ecology (e.g. Proud et al. 2012), phylogeography, and biogeography (e.g. Sharma and Giribet 2012).

By providing a database on harvestmen of French Guiana, regularly updated and publicly available, our goal is to provide a fast and efficient tool for sharing and tracking information on collected specimens. This database will be used for disseminating biodiversity information related to ongoing work on ecology and evolution of harvestmen in French Guiana. We also aim to promote best practices for recording and sharing biodiversity data within our research community. We believe this will make a valuable contribution to the global effort of sharing harvestmen database information (e.g. Merino-Sáinz et al. 2013).

## Project description

### Title

Harvestmen of French Guiana

### Personnel

Jérôme Murienne

### Study area description

Collecting trips have been conducted in various locations throughout French Guiana.

### Design description

This dataset was developed to determine the current distribution patterns of harvestmen species at the scale of French Guiana. Thus locations to be sampled were selected to maximize the geographical coverage. For some collecting trips (e.g. Saül 2013), a specific design was implemented to sample over three topographic units: hilltop, slope and seasonally-flooded bottomland.

### Funding

Data for this resource have been obtained within the framework of grants DIADEMA and PHYLOGUIANAS from the Labex CEBA (Laboratoire d'Excellence Center for the Study of Biodiversity in Amazonia). CEBA is funded by "Investissement d'Avenir" grant managed by the French National Research Agency (ANR) under grant number ANR-10-LABX-25-CEBA.

## Sampling methods

### Study extent

Study sites were located throughout French Guiana.

### Sampling description

The following techniques were used, however not all techniques were used on every collecting site and sampling design might not be similar among these sites.

Pitfall traps: 20 plastic cups with 70% ethanol were distributed within a 0.5 hectare plot and exposed for 72 hours.

Winkler: all the litter and superficial soil layer from 1 m^2^ was concentrated with the aid of a hand-sieve and sorted using a winkler apparatus during 2 days.

Litter manual sorting: all the litter and superficial soil layer from 1 m^2^ was concentrated with the aid of a hand-sieve and sorted by hand using a plastic square plate.

Beating: vegetation above knee-level was sampled using a 1 m^2^ beating sheet.

Sweeping: vegetation below knee-level was sampled using a sweep net.

Manual nocturnal ground search: samples are collected during the night on the ground or at the base of trunks.

Manual search in and under the dead wood. Samples are collected by searching in and under the dead wood pieces or the dead trunks on the ground.

### Quality control

GPS coordinates were obtained using a GPSmap 60CSx device or similar. Such devices report coordinates accuracy using the CEP50 (Circular Error Probability), meaning that there is only 50% probability that a reported position would be within a distance of X meters to the real position. Considering other sources of GPS errors (such as ionosphere delay and signal multi path) we estimate the accuracy of the coordinates to be around 30 meters at a 95% confidence level.

Initial specimens identifications to the family level (for all families) and genus level (for Agoristenidae, Stygnidae and Gonyleptidae) have been checked by Ricardo Pinto-da-Rocha. Validity of the taxonomic names used was checked against the Encyclopedia of Life (http://www.eol.org) and the GBIF backbone taxonomy (http://www.gbif.org/dataset/d7dddbf4-2cf0-4f39-9b2a-bb099caae36c). Future validity checks could use the "World Checklist of Opiliones species", once it is published in its entirety (Kury et al. 2014).

### Step description

After collection, samples are sorted and placed in individual tubes containing 95% ethanol with a unique identifier until further processing in the laboratory. Codes are based on locality, collection date, collection method followed by a unique number as in GF140308HC001-07 which indicates that the specimen was collected in French Guiana (GF) on 2014-03-08 (yyyy-mm-dd), and hand collected (HC). Collection method codes include sweep net (SN, SND or SNN), beating (BND or BNN), pitfall (PT), winkler (WK, WKD or WKN). When specimen collection was part of a larger field expedition, we kept the original codes that might not follow this rule (as in SL13-1208-OP01 collected in Saül).

**Figure 1.**
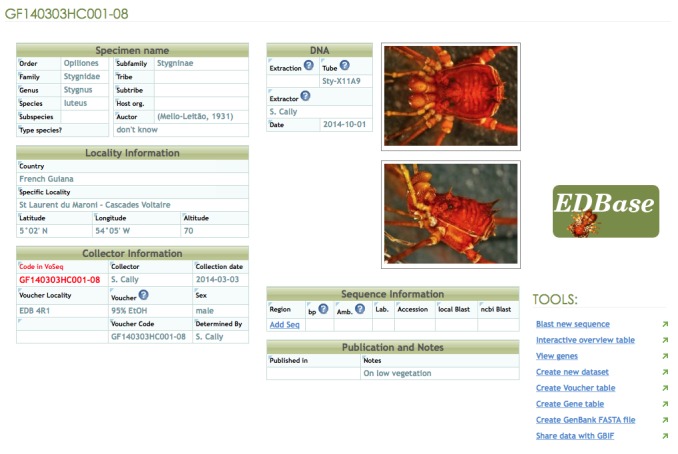
Example data entry of our online database (http://edbase.ups-tlse.fr with restricted access) holding the "Harvestmen of French Guiana" dataset. The database can be downloaded as a spreadsheet at: http://130.120.204.55:8080/ipt/resource.do?r=harvestmen_of_french_guiana or at: http://www.gbif.org/dataset/3c9e2297-bf20-4827-928e-7c7eefd9432c.

Collecting information for each specimen is databased in an online Voseq database (Peña and Malm 2012) hosted by the EDB laboratory (Fig. 1).

Specimens are photographed using an Olympus DP20 mounted on a Leica MZ16 binocular and pictures are stored in the database.

For each specimen, 1 or 2 legs are kept in a separate vial and stored at -20°C for further molecular investigation.

Specimens are initially curated at the EDB laboratory by J. Murienne and can be deposited in museums for further taxonomic study. For example, specimens of Sclerosomatidae have been sent as gift to the Museum of Comparative Zoology (MCZ) at Harvard University, Cambridge USA, to be investigated by Ana Lucia Tourinho. The current localisation of the specimens (either at EDB or in museums) can be found under the "disposition" field.

## Geographic coverage

### Description

**Figure 2.**
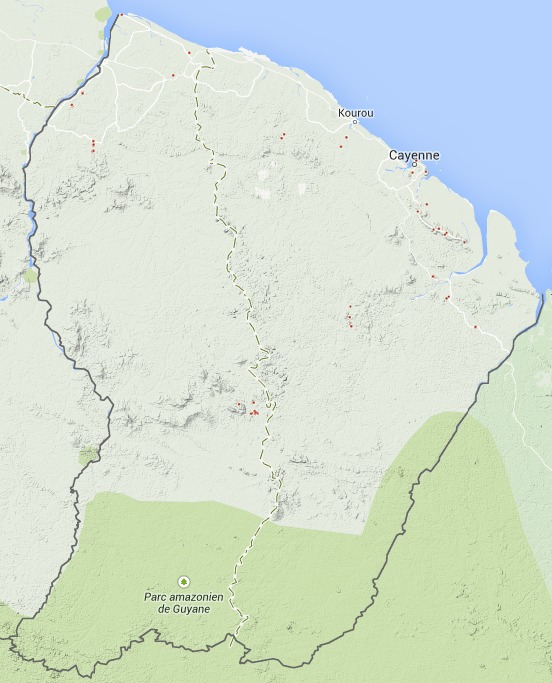
Geographical coverage of the dataset and collecting localities as of 2014.

The sampling area is delimited by the current administrative boundaries of the French Guiana territory (Fig. 2). To the East, the Oyapock River delimits the frontier with Brazil. To the West, the Maroni River delimits the frontier with Surinam. This is an important detail as the delimitation of the territory has not been constant throughout history and a large portion of northern Brazil was disputed between France and Brazil during the 19th century. Several species described or reported from French Guiana during the 19th century and early 20th century (see Kury 2003) are actually located in Brazil. Even though French Guiana is an overseas department and region of France, all occurrences have been recorded under the "French Guiana" country to comply with the ISO 3166-1 standard.

### Coordinates

2.09 and 5.85 Latitude; -51.53 and -54.61 Longitude.

## Taxonomic coverage

### Description

**Figure 3.**
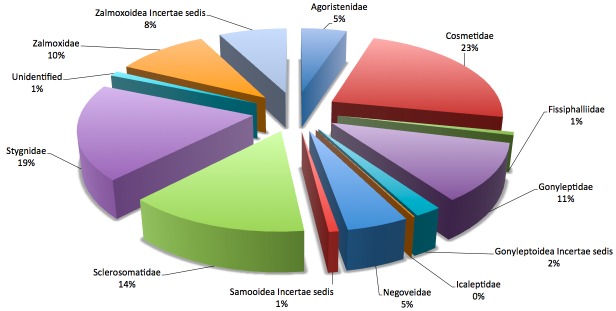
Taxonomic coverage at the family level of the "Harvestmen of French Guiana" dataset as of 2014.

This database is devoted to all harvestmen species inhabiting French Guiana. Little is known about the taxonomy of the group in French Guiana but some information are available in Kury (2003) for Laniatores and Benavides and Giribet (2013) for Cyphophthalmi. Identifications to the family level were conducted based on Kury and Pinto-da-Rocha (2002) and Pinto-da-Rocha et al. (2007)  (Fig. 3). Three families are here reported for the first time in French Guiana, namely Agoristenidae, Fissiphalliidae and Icaleptidae. Identifications of Stygnidae to the species level were conducted based on Pinto-da-Rocha (1997).

### Taxa included

**Table:** 

Rank	Scientific Name	Common Name
kingdom	Animalia	
phylum	Arthropoda	
class	Arachnida	
order	Opiliones	
family	Neogoveidae	
family	Sclerosomatidae	
family	Agoristenidae	
family	Stygnidae	
family	Cosmetidae	
family	Gonyleptidae	
family	Fissiphalliidae	
family	Zalmoxidae	
family	Icaleptidae	

## Temporal coverage

### Notes

2012-present

## Collection data

### Collection name

EDB arthropod collection

### Parent collection identifier

EDB specimen collection

### Specimen preservation method

Alcohol (95%)

## Usage rights

### Use license

Other

### IP rights notes

This work is licensed under a Creative Commons Attribution-NonCommercial 4.0 International Public License. http://creativecommons.org/licenses/by-nc/4.0/. Users of this resource should also comply with the CEBA data sharing agreement available here: www.labex-ceba.fr/assets/CEBA_Data_Sharing_Agreement_nov2013.pdf

## Data resources

### Data package title

Harvestmen of French Guiana

### Resource link

http://130.120.204.55:8080/ipt/resource.do?r=harvestmen_of_french_guiana

### Alternative identifiers

http://www.gbif.org/dataset/3c9e2297-bf20-4827-928e-7c7eefd9432c

### Number of data sets

1

### Data set 1.

#### Data set name

Harvestmen of French Guiana

#### Data format

Darwin Core Archive format

#### Number of columns

29

#### Character set

UTF-8

#### Data format version

1.0

#### Description

**Data set 1.** 

Column label	Column description
occurrenceID	An identifier for the Occurrence (as opposed to a particular digital record of the occurrence). In the absence of a persistent global unique identifier, construct one from a combination of identifiers in the record that will most closely make the occurrenceID globally unique. See also http://rs.tdwg.org/dwc/terms/index.htm#occurrenceID For line numbers you can specify an optional non-numerical suffix to be appended to the id. This is useful to generate unique identifiers when mapping the same source multiple times.
institutionCode	The name (or acronym) in use by the institution having custody of the object(s) or information referred to in the record.
collectionCode	The name, acronym, coden, or initialism identifying the collection or data set from which the record was derived.
basisOfRecord	The specific nature of the data record - a subtype of the dcterms:type. Recommended best practice is to use a controlled vocabulary such as the Darwin Core Type Vocabulary (http://rs.tdwg.org/dwc/terms/type-vocabulary/index.htm).
catalogNumber	An identifier (preferably unique) for the record within the data set or collection.
occurrenceRemarks	Comments or notes about the Occurrence.
recordedBy	A list (concatenated and separated) of names of people, groups, or organizations responsible for recording the original Occurrence. The primary collector or observer, especially one who applies a personal identifier (recordNumber), should be listed first.
sex	The sex of the biological individual(s) represented in the Occurrence. Recommended best practice is to use a controlled vocabulary.
preparations	A list (concatenated and separated) of preparations and preservation methods for a specimen.
disposition	The current state of a specimen with respect to the collection identified in collectionCode or collectionID. Recommended best practice is to use a controlled vocabulary.
otherCatalogNumbers	A list (concatenated and separated) of previous or alternate fully qualified catalog numbers or other human-used identifiers for the same Occurrence, whether in the current or any other data set or collection.
associatedMedia	A list (concatenated and separated) of identifiers (publication, global unique identifier, URI) of media associated with the Occurrence.
associatedReferences	A list (concatenated and separated) of identifiers (publication, bibliographic reference, global unique identifier, URI) of literature associated with the Occurrence.
associatedTaxa	A list (concatenated and separated) of identifiers or names of taxa and their associations with the Occurrence.
eventDate	The date-time or interval during which an Event occurred. For occurrences, this is the date-time when the event was recorded. Not suitable for a time in a geological context. Recommended best practice is to use an encoding scheme, such as ISO 8601:2004(E).
country	The name of the country or major administrative unit in which the Location occurs.
locality	The specific description of the place. Less specific geographic information can be provided in other geographic terms (higherGeography, continent, country, stateProvince, county, municipality, waterBody, island, islandGroup). This term may contain information modified from the original to correct perceived errors or standardize the description.
verbatimLocality	The original textual description of the place.
verbatimElevation	The original description of the elevation (altitude, usually above sea level) of the Location.
decimalLatitude	The geographic latitude (in decimal degrees, using the spatial reference system given in geodeticDatum) of the geographic center of a Location. Positive values are north of the Equator, negative values are south of it. Legal values lie between -90 and 90, inclusive.
decimalLongitude	The geographic longitude (in decimal degrees, using the spatial reference system given in geodeticDatum) of the geographic center of a Location. Positive values are east of the Greenwich Meridian, negative values are west of it. Legal values lie between -180 and 180, inclusive.
identifiedBy	A list (concatenated and separated) of names of people, groups, or organizations who assigned the Taxon to the subject.
scientificName	The full scientific name, with authorship and date information if known. When forming part of an Identification, this should be the name in lowest level taxonomic rank that can be determined. This term should not contain identification qualifications, which should instead be supplied in the IdentificationQualifier term.
order	The full scientific name of the order in which the taxon is classified.
family	The full scientific name of the family in which the taxon is classified.
genus	The full scientific name of the genus in which the taxon is classified.
specificEpithet	The name of the first or species epithet of the scientificName.
infraspecificEpithet	The name of the lowest or terminal infraspecific epithet of the scientificName, excluding any rank designation.
scientificNameAuthorship	The authorship information for the scientificName formatted according to the conventions of the applicable nomenclaturalCode.
